# Molecular characterization and protective efficacy of vacuolar protein sorting 29 from *Eimeria tenella*

**DOI:** 10.3389/fcimb.2023.1205782

**Published:** 2023-07-04

**Authors:** Tuan-yuan Shi, Tian-en Li, Yun Hao, Hong-chao Sun, Yuan Fu, Wen-chao Yan, Li-li Hao

**Affiliations:** ^1^ Institute of Animal Husbandry and Veterinary Medicine, Zhejiang Academy of Agricultural Sciences, Hangzhou, Zhejiang, China; ^2^ College of Animal Science and Technology, Henan University of Science and Technology, Luoyang, Henan, China; ^3^ College of Animal & Veterinary Sciences, Southwest Minzu University, Chengdu, Sichuan, China

**Keywords:** *Eimeria tenella*, Vacuolar protein sorting 29, recombinant protein, subcellular localization, protective efficacy

## Abstract

**Introduction:**

Vacuolar protein sorting 29 (VPS29) is a core component of the retromer-retriever complex and is essential for recycling numerous cell-surface cargoes from endosomes. However, there are no reports yet on VPS29 of *Eimeria* spp.

**Methods:**

Here, we cloned and prokaryotically expressed a partial sequence of *Eimeria tenella* VPS29 (EtVPS29) with RT-PCR and engineered strain of *Escherichia coli* respectively. The localization of the VPS29 protein in *E. tenella* sporozoites was investigated with immunofluorescence (IFA) and overexpression assays. And its protective efficacy against *E. tenella* infection was investigated in chickens with the animal protection test.

**Results:**

An EtVPS29 gene fragment with an ORF reading frame of 549 bp was cloned. The band size of the expressed recombinant protein, rEtVPS29, was approximately 39 kDa and was recognized by the chicken anti-*E. tenella* positive serum. EtVPS29 protein was observed widely distributing in the cytoplasm of *E. tenella *sporozoites in the IFA and overexpression assays. rEtVPS29 significantly increased average body weight gain and decreased mean lesion score and oocyst output in chickens. The relative weight gain rate in the rEtVPS29-immunized group was 62.9%, which was significantly higher than that in the unimmunized and challenged group (P < 0.05). The percentage of reduced oocyst output in the rEtVPS29 immunized group was 32.2%. The anticoccidial index of the rEtVPS29-immunized group was 144.2. Serum ELISA also showed that rEtVPS29 immunization induced high levels of specific antibodies in chickens.

**Discussion:**

These results suggest that rEtVPS29 can induce a specific immune response and is a potential candidate for the development of novel vaccines against *E. tenella* infections in chickens.

## 1 Introduction

Chicken coccidiosis caused by *Eimeria* spp., an intracellular apicomplexan parasite, leads to an estimated loss of 3.5 billion USD annually worldwide (Blake et al., 2020). *Eimeria tenella* is the most pathogenic and common of the seven known chicken-infecting species (Blake et al., 2021). *Eimeria tenella* infection is not age-restricted and mainly targets cecal epithelial cells, leading to necrosis of the intestinal epithelial cells and injury to the intestinal mucosa. Bloody stool is a common clinical symptom. Mild infections can affect the growth, development, and production performance of chickens, whereas severe infections can cause the mass death of chickens (Chapman et al., 2013; Blake et al., 2021). Traditional strategies for the prevention and control of chicken coccidiosis rely primarily on drugs and live vaccines. However, these measures have been restricted for several reasons, including the frequent emergence of drug-resistant strains, growing consumer attention to food safety, virulence reversion, and production difficulties. Therefore, novel and cost-effective anticoccidial control strategies need to be developed (Peek and Landman, 2011; Martins et al., 2022). The key to develop novel vaccines is the identification of efficient antigen proteins against coccidiosis (Gupta and Shukla, 2016).

Vacuolar protein sorting 29 (VPS29) is a core component of the retromer-retriever complex and is essential for recycling numerous cell-surface cargoes from endosomes (Baños-Mateos et al., 2019). The retromer and retriever complexes consist of cargo-selective subunits (VPS35/VPS35L, VPS26/VPS26C, and VPS29) and sorted nexin subunits (VPS5 and VPS17) (Gershlick and Lucas, 2017; McNally et al., 2017). The main function of the retromer and retriever complexes is to interact with the endosomal membrane and sort multiple transmembrane cargoes such as heterodimeric integrin α5β1, low-density lipoprotein receptor-related protein 1 (LRP1), amyloid precursor protein (APP), and epidermal growth factor receptor (EGFR), which are then transported back from the endosome to the Golgi apparatus or plasma membrane to recycle the receptors (Mukadam and Seaman, 2015). The absence of VPS29 can disrupt the integrity of the complex, affecting transport between the endosome and the plasma membrane or Golgi apparatus, as well as other physiological processes within the cell (Kang et al., 2012; McNally et al., 2017).

The structure and function of VPS29 have been reported in plant (Jaillais et al., 2007), yeast (Iwaki et al., 2006), and anima (Mitsui et al., 2011) cells. However, there have been no reports on *E. tenella* VPS29. In the present study, EtVPS29 was cloned and expressed prokaryotically. An animal protection test was performed to evaluate the protective efficacy of rEtVPS29 against *E. tenella* infection, providing a foundation for the development of novel vaccines against chicken coccidiosis.

## 2 Materials and methods

### 2.1 Ethics and animals

All protocols were approved by the Animal Care Committee of Zhejiang Academy of Agricultural Sciences in accordance with the recommendations of the National Institutes of Health Guide for the Care and Use of Laboratory Animals (Ethics protocol No. 2020ZAASLA014).

One-day-old specific pathogen-free (SPF) chickens were purchased from a local poultry breeding center (Xiaoshan, Hangzhou, China). These animals were raised in an insulation house (30–37°C) and offered forage (without any anticoccidial drugs) and cold boiled water; the forage was baked at 70°C in an oven for at least 2 h before feeding to the chickens.

### 2.2 Parasite propagation and purification

The Beijing strain of *E. tenella* gained from professor Xun Suo of China Agricultural University was used in the following experiments. The strain were propagated by passage in 2-week-old coccidia-free chickens. Unsporulated oocysts were collected from fresh feces 7-9 days after oral infection with 1× 10^4^ sporulated oocysts per chicken. Procedures for isolation, purification and sporulation of oocysts of *E. tenella* have been described previously (Long et al., 1976). Sporozoites were excysted with a modified excystation buffer (PBS pH 7.6, containing 0.75% trypsin and 10% chicken bile extract) and purified by DE-52 anion-exchange chromatography (Schmatz et al., 1984).

### 2.3 Cloning and bioinformatics analysis of EtVPS29 gene

According to public information on the VPS29 protein coding gene (GeneID: 25250978) of the *E. tenella* Houghton strain, a putative partial gene sequence with 586 bp nucleotides coding 195 amino acids, from database of National Center for Biotechnology Information (NCBI), specific primer pairs were designed using Primer 5.0 software. The designed sequences were as follows: upstream primer 5’-GGATCCATGGGATTGACTGGCTTTTTT-3’ (with the *Bam*HI restriction site underlined) and the downstream primer 5’-AAGCTTGAGTCTCCCTTACTCCTC-3’ (with the *Hin*dIII restriction site underlined). The primers were synthesized by Hangzhou Youkang Biotechnology Co., Ltd.

The total RNA of the sporozoites was extracted and subjected to RT-PCR to determine the presence of the EtVPS29 gene. The reaction comprised pre-denaturation at 94°C for 5 min, 35 cycles of denaturation at 94°C for 30 s, annealing at 58°C for 30 s, and extension at 72°C for 1 min, and final extension at 72°C for 10 min. After sequencing, the amino acid sequence was analyzed to predict the molecular weight (MW) and isoelectric point using the ExPASy ProtParam tool and the signal peptide and transmembrane region of the gene using the SignalP 5.0 Server and TMHMM 2.0 Server. Phylogenetic tree was constructed using MegAlign of Lasergene software. All the reference sequences of other species are downloaded from NCBI and listed in **Table 1**.

**Table 1:** Genbank reference sequences used in construction of the phylogenetic tree.

Species	Source/location	Genbank accession no.
*Eimeria tenella*	Houghton strain from chicken, United kingdom	XP_013234043.1
*Eimeria necatrix*	Houghton strain from chicken, United kingdom	XP_013439298.1
*Cyclospora cayetanensis*	Stool of Homo sapiens, Nepal	XP_022589174.1
*Toxoplasma gondii*	ME49 strain, America	XP_018638204.1
*Neospora caninum*	Liverpool strain, United kingdom	XP_003882471.1
*Besnoitia besnoiti*	Bb-Ger1 strain from cattle, Germany	XP_029215183.1
*Plasmodium vivax*	Brazil I from Homo sapiens, America	KMZ83952.1
*Cryptosporidium bovis*	Stool of cattle, China	KAH7650341.1
*Gallus gallus*	/	NP_001264434.1
*Homo sapiens*	/	NP_001269080.1
*Canis lupus familiaris*	/	NP_001273799.1
*Equus caballus*	/	XP_001495149.1
*Bos taurus*	/	NP_001029627.1
*Oryctolagus cuniculus*	/	XP_051682746.1
*Caenorhabditis elegans*	Bristol N2 strain, America	NP_001022987.1
*Saccharomyces cerevisiae*	YJM1336 strain, Italy	AJV31545.1

”/” indicates no informations.

### 2.4 Expression and purification of rEtVPS29

The EtVPS29 fragment was cloned and ligated into the pMD19-T vector. The cloned plasmid pMD19-EtVPS29 and the pET32a plasmid vector were double-digested and ligated using T4 ligase. The ligated plasmids were transfected into competent BL21(DE3) *Escherichia coli* cells. Once the OD600 value of the positive bacteria reached 0.6–0.8, IPTG at a final concentration of 1 mM was added to induce expression and the cells were incubated at 37°C and 200 rpm for 4 h. The induced bacterial culture was centrifuged at 8000 rpm for 5 min. The supernatant was discarded and the collected bacterial cells were resuspended in 0.01 M PBS, washed thrice, and then suspended in 0.01 M PBS and kept on ice.

The bacterial suspension was subjected to ultrasonic crushing at 4°C, followed by centrifugation at 12,000 rpm for 10 min to separate the supernatant and precipitate. The precipitate and supernatant were subjected to SDS-PAGE to confirm the presence of the recombinant protein. The recombinant protein was purified by nickel ion affinity chromatography. The purified protein was dialyzed with 0.01 M PBS, and the purification efficiency was analyzed by SDS-PAGE.

### 2.5 Western blot analysis of rEtVPS29

SDS-PAGE was performed using 6 μg of the recombinant protein or sporozoite protein. The sporozoite protein was prepared as follows: 5×10^7^ purified sporozoites were lysated in 500 μL RIPA lysis buffer (Solarbio, China) and treated with ultrasonic crushing for 10 sec at 4°C, followed by centrifugation at 12,000 rpm for 10 min to separate the supernatant. Protein concentration of the supernatant was determined with a BCA protein assay kit (Beyotime, China) and used in the SDS-PAGE. Following electrophoresis, the gel, NC membrane, and filter paper were appropriately sized, balanced in the transfer solution, and transferred using a 200 mA transfer system for 15 min. After transfer, the membrane was examined *via* Ponceau staining and washed with 0.01 M PBS three times, with each wash lasting 5 min. The membrane was then coated with 5% skim milk powder for 2 h and the coating liquid was discarded before washing. Primary antibodies (anti-*E. tenella*-positive chicken serum and -negative chicken serum) were added at a 1:150 dilution and incubated overnight at 4°C. After washing with 0.01 M PBS, the membrane was incubated with horseradish peroxidase (HRP)-labeled rabbit anti-chicken IgG secondary antibody at a 1:6000 dilution for 2 h. The membrane was then washed with 0.01 M PBS thrice, followed by the addition of chromogenic solution according to instructions of the ECL ultrasensitive luminescence kit (Sangon Biotech, China).

### 2.6 EtVPS29 localization by indirect immunofluorescence assay

Six-week-old SPF mice were immunized with a predetermined concentration of rEtVPS29 mixed and fully emulsified with Freund’s complete adjuvant or Freund’s incomplete adjuvant at a 1:1 ratio, and administered through a subcutaneous multipoint injection in the neck. The dose of the first immunization was 100 μg of recombinant protein per mouse, followed by a second and third immunization with 50 μg per mouse at 14-day intervals. Seven days after the third immunization, the eyeballs were removed and blood was collected. The collected blood was allowed to clot at 37°C for 1 h and then refrigerated at 4°C overnight. The following day, the serum was separated by centrifugation at 3000 rpm for 5 min and stored in a refrigerator at −80°C. The titer of the polyclonal antibody was determined by indirect ELISA using rEtVPS29 as the antigen, rEtVPS29-immunized mouse serum as the primary antibody, and HRP-labeled goat anti-mouse IgG as the secondary antibody. H2SO4 was used to terminate color development and OD450 values were measured using a SpectraMax M microplate reader (Molecular Device, USA).

Purified sporozoites (1×10^7^) were fixed with 4% paraformaldehyde for 30 min, permeabilized with 0.2% Triton-X-100 for 30 min, and washed thrice with 0.01 M PBS. Subsequently, the sporozoites were blocked with 5% BSA at 37°C for 1 h and washed thrice with 0.01 M PBS. The prepared polyclonal antiserum was diluted 1:800 and added dropwise to the sporozoites, which were then incubated at room temperature for 1 h and washed thrice with 0.01 M PBS. The fluorescent secondary antibody (Alexa Fluor^®^ 488-labeled goat anti-mouse IgG) was diluted 1:500 and dropped onto the sporozoites, followed by incubation at room temperature for 1 h, and washing three times with 0.01 M PBS. For staining the nucleus, the sporozoites were incubated with DAPI (diluted to 5 μg/ml) for 2 min and washed three times with 0.01 M PBS. The sporozoites were then observed using a fluorescence microscope (SOPTOP, China) with 1000× magnification and 100× objective lens. And pictures were acquired with a image processing software (ImageView, China).

### 2.7 Localization analysis of overexpressed VPS29 fusion with fluorescent protein

A plasmid expression vector from *E. tenella, Et*H4-2EYFP-Actin was digested using the restriction enzymes *Spe*I and *Age*I to gain a linearized *Et*H4-EYFP-Actin vector. The VPS29 fragment carrying the restriction enzyme sites for *Spe*I and *Age*I was amplified using PCR and then ligated into the linearized *Et*H4-EYFP-Actin vector to construct plasmid *Et*H4-VPS29-EYFP-Actin.

The *Et*H4-VPS29-EYFP-Actin plasmid was extracted in large quantities using a plasmid extraction kit (Solarbio Life Sciences, China). After determining the DNA concentration, the plasmid was linearized by overnight single-enzyme digestion with *Bam*HI. Following this, 10 μL of 3 M sodium acetate was added to the plasmid and mixed with 200 μL of pre-cooled anhydrous ethanol before being stored at −20°C for 30 min. The supernatant was then removed after centrifugation at 12000 rpm for 10 min, and the remaining DNA was washed twice with 75% ethanol before being dissolved and precipitated with 100 μL of complete electroporation solution. The concentration of DNA was measured to ensure that the total content was not less than 50 μg. To prepare for electroporation, 5 × 10^6^ sporozoites were washed thrice with and resuspended in the complete electroporation solution. Then, we prepared 800 μL of an electroporation mixture, consisting of 50 μg of linearized *Et*H4-VPS29-EYFP-Actin plasmid, 5 × 10^6^ sporozoites, and 100 U of *Bam* HI, with the remaining volume made up with complete electroporation solution. Electroporation was performed using a Gene Pulser X CellTM (Bio-Rad, USA) at 2000 V and 25 µF with a pulse time of about 0.3 ms. After electroporation, the mixture was left undisturbed at room temperature for at least 20 min. Electroporated sporozoites were inoculated into five chickens *via* the cloaca. Oocysts from feces excreted between 7 and 10 days post-inoculation were collected using the saturated NaCl flotation method and sporulated in 2.5% potassium dichromate at 28°C. Sporulated oocysts were purified with 13% sodium hypochlorite solution and then washed with sterilized PBS. Transgenic oocysts were screened by fluorescence-activated cell sorting using a MoFlo Cell Sorter (Dako Cytomation, Fort Collins, CO, USA). The sorted fluorescent oocysts were propagated in coccidia-free chickens. Stable transfection of *E. tenella* was obtained after several sorting and propagation cycles in coccidia-free chickens. The expression of the VPS29 fusion fluorescent protein was confirmed using fluorescence microscopy (SOPTOP, China).

### 2.8 Immune and challenge infection

As shown in **Table 2**, 30 one-week-old chickens were randomly divided into three groups of 10 chickens with equal mean body weights. The experimental group was immunized with 100 μg EtVPS29 recombinant protein emulsified in complete Freund’s adjuvant as a 1:1 mixture per chicken by subcutaneous injection on the neck. The chickens in the challenged and unchallenged control groups were injected with PBS emulsified with complete Freund’s adjuvant. The second and third immunizations were performed at a 14-day interval using the same dose as the primary immunization. Seven days after the third immunization, blood samples were collected to isolate serum, which was used for antibody detection. All chickens, except those in the unchallenged control group, were orally challenged with 2×10^4^ sporulated oocysts of *E. tenella*. Chickens in the unchallenged control group were orally administered equal volumes of PBS. Fecal samples from each group were collected separately from the 6th to the 9th day post-challenge. On the 9th day after the challenge, all chickens were weighed and slaughtered. The cecal content of each group was collected separately. The average body weight gain, oocyst decrease ratio, lesion score, and anticoccidial index (ACI) were calculated. Body weight gain was determined by subtracting the body weight at the time of challenge from that at the end of the experiment. Cecal lesion scores were determined on a graded scale from 0 (normal) to 4 (severe), as described by Johnson and Reid (1970). Oocysts per gram of fecal sample and cecal content were counted using McMaster’s technique. The oocyst decrease ratio was calculated as follows: (number of oocysts from the positive control chickens–immunized chickens)/positive control chickens × 100%. ACI is a synthetic criterion for determining the anticoccidial effect and is calculated as follows: (relative rate of weight gain + survival rate) × (lesion value + oocyst value). An ACI greater than 180 was deemed excellent, 160 < ACI ≤ 180 was considered good, an ACI between 120 and 160 was generally effective, and an ACI less than 120 was considered invalid.

**Table 2:** Groups of experimental animal and immunization program.

Groups	Number	immune dosage (total volume 0.2 mL per chicken)	challenge dosage (sporulated oocysts per chicken)
unchallenged control	10	PBS+Freund’s adjuvant	0
challenged control	10	PBS+Freund’s adjuvant	2×10^4^
rEtVPS29 immunized	10	100 µg rEtVPS29+Freund’s adjuvant	2×10^4^

### 2.9 Detection of specific antibody levels against EtVPS29

The specific antibody level against EtVPS29 in the immunized chicken serum was detected by ELISA. To conduct ELISA, the recombinant protein was diluted to a concentration of 1 μg/μL with a coating solution and added to an ELISA reaction plate, which was then incubated at 4°C overnight. The coated plate was subsequently dried and washed three times with the washing solution. To prevent nonspecific binding, a blocking solution was added to the plate and incubated at 37°C for 2 h. After drying and washing the plate again, the primary antibody (immunized chicken serum or unimmunized chicken serum collected above) was diluted using a gradient dilution of 1:100 to 1:12800 and added to the plate, followed by incubation at 37°C for 1 h. The plate was then dried and washed three times with the washing solution. The secondary antibody, HRP-labeled goat anti-chicken IgG, was diluted with diluent at a concentration of 1:10000 and added to the plate, followed by incubation at 37°C for 1 h. After drying and washing the plate again, a colored solution was mixed in proportion and added to the wells, followed by incubation at 37°C for 20 min. After adding the termination solution, OD450 was measured using a microplate reader.

### 2.10 Statistical analysis

All data were analyzed using GraphPad Prism software (version 8.0), and the results are expressed as the arithmetic mean ± standard deviation. At least three independent experiments were conducted and one-way analysis of variance (ANOVA) was used to analyze the data. Statistical significance was set at *p* < 0.05.

## 3 Results

### 3.1 Construction of EtVPS29 recombinant expression plasmid

**Figure 1:**
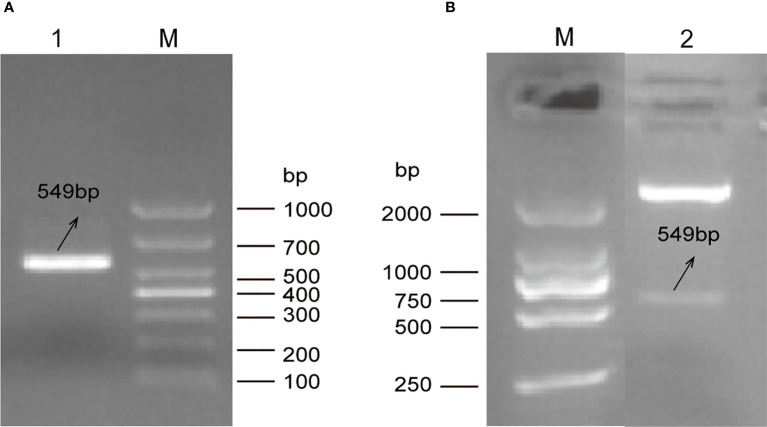
Construction of prokaryotic expression plasmid pET32a (+)-EtVPS29. M: DNA marker. **(A)** Amplification of EtVPS29 fragment *via* PCR. Lane 1: Product of the PCR. **(B)** Double digestion identification of pET32a (+) -EtVPS29. Lane 2: Product from the recombinant plasmid pET32a(+)-EtVPS29 digested with *Bam*HI and *Hin*dIII restriction enzymes.

A single electrophoretic band of DNA with a size between 500 and 700bp was amplified from the total RNA of *E. tenella* Beijing strain using RT-PCR (**Figure 1A**). The sequence length of amplified fragment was 549 bp encoding 183 amino acids. Comparing with full length of the putative *E. tenella* VPS29 sequence (585bp) in NCBI, 36 bp nucleotides missed in the 3’ -terminal. Sequence analysis indicated that the amplified fragment was 100% identical to the VPS29 protein-coding gene (Gene ID:25250978) of *E. tenella* Houghton strain. The recombinant expression plasmid, pET32a (+)-EtVPS29, was successfully constructed (**Figure 1B**).

### 3.2 VPS29 gene is highly conserved within apicomplexan parasites

**Figure 2:**
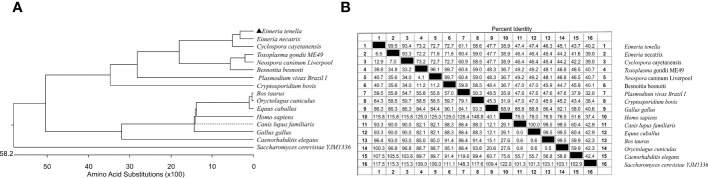
Phylogenetic tree and homology analysis basing on VPS29 genes. **(A)** Phylogenetic tree basing on VPS29 genes from different species. **(B)** Similarity and differences between sequences of VPS29 genes from different species.

The predicted relative molecular weight was 20.192 kDa and the isoelectric point was 5.045. No signal peptides or transmembrane structures were identified. The VPS29 gene sequence from *E. tenella* shared 99.5% percent identity with the sequence from *E. necatrix*, but shared 47.7% percent identity with its host–*Gallus gallus*. Results of the alignment analysis revealed that VPS29 is highly conserved within apicomplexan parasites, especially within *Eimeria* spp. and there are obvious differences between apicomplexan parasites and higher animal cells (**Figures 2A, B**).

### 3.3 Generation of recombinant rEtVPS29 protein

**Figure 3:**
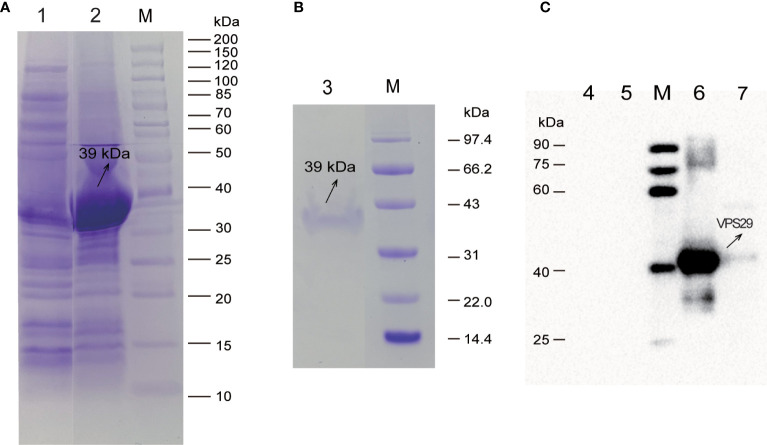
Expression, purification, and western blot analysis of rEtVPS29. M: Protein Marker. **(A)** The expression of rEtVPS29 was analyzed by SDS-PAGE. Lane 1: Supernatant from induced pET32a (+) -EtVPS29/BL21 (DE3) bacteria, Lane 2: Precipitation from induced pET32a(+)-EtVPS29/BL21 (DE3) bacteria. **(B)** Analysis of rEtVPS29 by SDS-PAGE. Lane 3: Purified rEtVPS29 protein. **(C)** Western blot analysis of rEtVPS29. Lane 4-5: negative control, Lane 6: VPS29 recombinant protein, Lane 7: Total proteins from *E. tenella* sporozoites.

The expression level of rEtVPS29 was the highest when the final concentration of IPTG was 1 mM and the induction temperature was 37°C for 4 h (200 rpm). The molecular weight of rEtVPS29 was approximately 39 kDa, and it existed as inclusion bodies (**Figure 3A**). The recombinant protein was purified using nickel ion affinity chromatography (**Figure 3B**). Furthermore, western blot analysis (**Figure 3C**) revealed that a specific antibody against EtVPS29 was produced in the anti-*E. tenella*-positive chicken serum, and the antibody could react with both the recombinant protein and whole parasite proteins. Western blotting results showed that the positive band size was consistent with the theoretical size, and the negative chicken serum did not react with the recombinant protein or whole parasite proteins.

### 3.4 EtVPS29 protein is widely distributed in the cytoplasm of *E. tenella*

**Figure 4:**
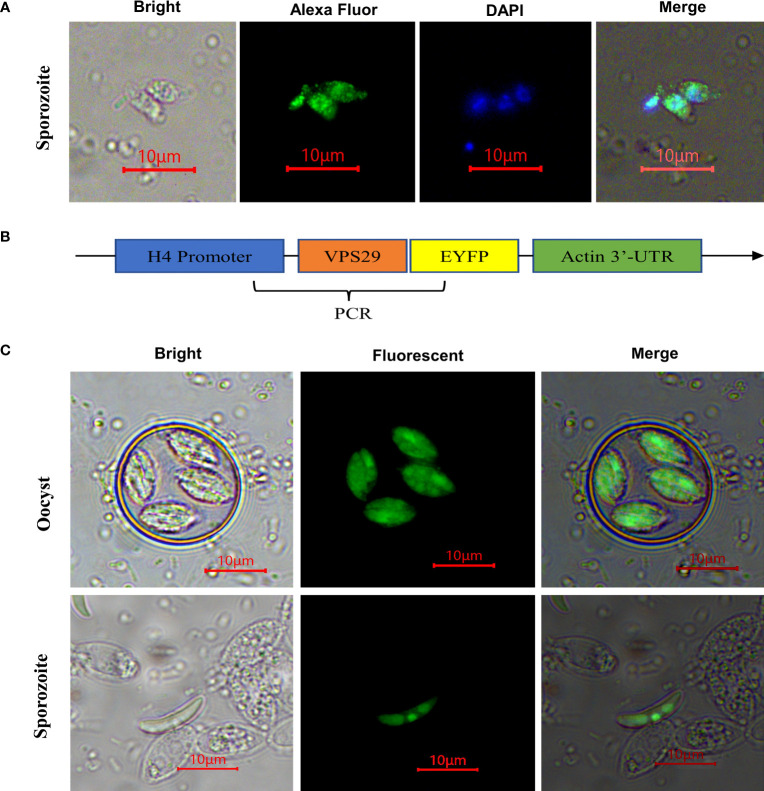
Localization of EtVPS29 in sporozoites of *Eimeria tenella*. **(A)** Localization analysis by immunofluorescence assay. Bri: bright field image, AF488: Alexa Fluor 488-labeled Goat Anti-Mouse IgG immunofluorescence staining image, DAPI: image of DAPI-stained nucleus, Mer: merged image. Scale bar, 10 μm. **(B)** EtH4-VPS29-EYFP-Actin plasmid structure diagram. **(C)** Localization analysis using overexpressed VPS29 fused with fluorescent protein. Bri: bright field image, Flu: fluorescent field image, Scale bar, 10 μm.

Indirect immunofluorescence results indicated EtVPS29 was distributed throughout the cytoplasm of *E. tenella* sporozoites (**Figure 4A**). In addition, to obtain transgenic *E. tenella* expressing EtVPS29 fused with yellow fluorescent protein, an *Et*H4-VPS29-EYFP-Actin plasmid expression vector was constructed (**Figure 4B**). EtVPS29 transgenic *E. tenella* was constructed through electrotransfection of *E. tenella* sporozoites, followed by the screening of passages in animals. Fluorescence microscopy showed that EtVPS29 was mainly expressed in the cytoplasm of *E. tenella* sporozoites, particularly in the nucleus, apical secretory organelles, and refractive bodies (**Figure 4C**). These findings were consistent with those obtained through indirect immunofluorescence localization.

### 3.5 rEtVPS29 shows immune protective efficacy against *E. tenella* infection

Chickens in the challenged control group exhibited severe clinical symptoms, such as depression and bloody stool on the 5^th^ day after the challenge, while those in the rEtVPS29-immunized group showed mild symptoms. The average weight gain of the rEtVPS29-immunized group was significantly higher than that of the challenged control group (*P* < 0.05), with a relative weight gain of 62.9%. The mean oocyst output in the rEtVPS29-immunized group was (2.06 ± 0.45) × 10^6^ per chicken, while that in the challenged control group was (3.04 ± 0.74) × 10^6^. Oocyst output was not observed in the unchallenged control group. Compared to the challenged control group, oocyst excretion in the rEtVPS29-immunized group was significantly reduced, with an oocyst reduction rate of 32.2% (*P* < 0.05). The cecal lesion score of the rEtVPS29-immunized group (1.86 ± 0.89) was lower than that of the challenged control group (2.57 ± 0.53). No lesions were observed in unchallenged controls. The ACI of the rEtVPS29-immunized group was 144.2, whereas that of the challenged control group was 102.9 (**Table 3**).

**Table 3:** Protective efficacy of rEtVPS29 against *Eimeria tenella* infection.

Groups	Mean body weight gain (g)	Relative body weight gain rate (%)	Oocyst output per chicken (×10^4^)	Reduced percentage of oocyst excretion (%)	Survival rate (%)	Mean lesion scores	ACI
unchallenged control	73.57±14.15_a_	100	0	100	100	0	200
challenged control	26.57±6.503_b_	36.6	3.04±0.74_a_	0	100	2.57±0.53_a_	102.9
rEtVPS29 immunized	49.86±10.48_a_	62.9	2.06±0.45_b_	32.2	100	1.86±0.89_a_	144.2

Data are presented as mean ± standard deviation. In each column, significant difference (P<0.05) between two data is indicated with different small letters (_a, b_).

**Figure 5:**
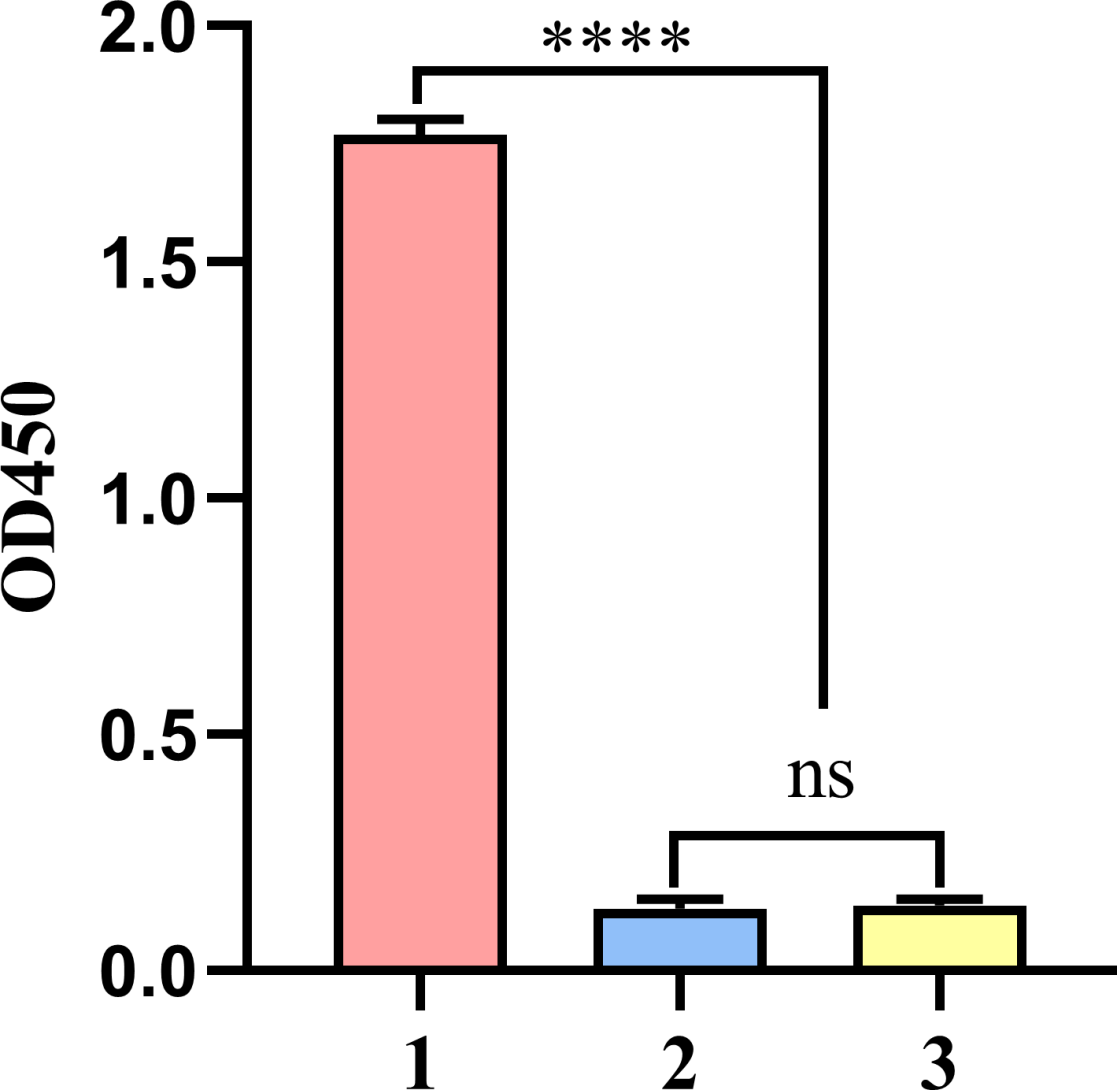
Anti-rEtVPS29 specific antibody level in chicken serum at the dilution of 1:12800. (1) Serum antibody levels in chickens immunized with rEtVPS29 (n=4). (2) Serum antibody level of chickens in non-immune non-challenged group (n=4). (3) Serum antibody level of chickens in non-immune challenge group (n=4). (n: number of chickens per group; ****P < 0.0001, ns, no significant difference).

In addition, the ELISA results indicated that rEtVPS29 immunization induced high levels of specific antibodies in chickens (**Figure 5**).

## 4 Discussion

VPS29 is an essential component of the retromer-retriever complex and plays an important role in recycling numerous transmembrane proteins from endosomes (Gershlick and Lucas, 2017; Baños-Mateos et al., 2019). It has been identified in many eukaryotes, including yeast, nematodes, humans, *Drosophila*, *Arabidopsis*, *Plasmodium falciparum*, and *Toxoplasma gondii* (Seaman et al., 1997; Damen et al., 2006; Nodzynski et al., 2013; Lorenowicz et al., 2014; Maruzs et al., 2015; Iqbal et al., 2016; Sangaré et al., 2016). However, there are currently no reports on the characteristics of *E. tenella* VPS29. In the present study, the VPS29 gene sequence of *E. tenella* was identified, cloned, and expressed. Cluster analysis revealed that VPS29 is highly conserved within apicomplexan parasites, particularly within *Eimeria* spp., and shows obvious differences between apicomplexan parasites and higher animal cells. We speculated that *Eimeria* VPS29 protein may have other mysterious cellular functions. Studies in *T. gondii* have shown that VPS29 plays roles not only in recycling transmembrane proteins from endosomes to the Golgi and plasma membrane, but also in parasite integrity, secretory organelle biogenesis, maintenance of a multiple ligand-binding transporter, and host infection (Sangaré et al., 2016). Unlike other eukaryotic cells, the Apicomplexa possess a highly complex endomembrane system, including the nuclear envelope, endoplasmic reticulum, Golgi, lysosomes, and related vacuoles, endosomes, and unique apicomplexan secretory organelles (micronemes, rhoptries, and dense granules), which are located in the cytoplasm (Wiser, 2021). Our results also indicated that VPS29 is widely present in the cytoplasm of *E. tenella* sporozoites. This is consistent with previous reports on *Plasmodium* (Iqbal et al., 2016). These results suggest that the conservation of and differences in VPS29 among species are closely related to their functions. *Eimeria tenella* VPS29 may have similar functions with that of the *T. gondii*.

Currently, there are no recombinant subunit vaccines available for chicken coccidiosis. A key prerequisite for novel vaccine development is the screening for highly efficient antigens. Approximately 6700 genes have been reported to be expressed across the four developmental life stages (unsporulated oocysts, sporulated oocysts, sporozoites, and merozoites) of *E. tenella* (Reid et al., 2014). A number of novel antigens, including parasite surface proteins such as Immune Mapped protein-1 and apical membrane antigen-1, and parasite internal antigens, such as microneme (MIC2/MIC3/MIC8), rhoptry (ROP17/ROP30/ROP35), and refractive proteins (SO7) (Dalloul et al., 2005; Konjufca et al., 2008; Huang et al., 2018; Liu et al., 2020; Ma et al., 2021; Zhao et al., 2021; Bingxiang et al., 2022; Liu et al., 2022; Wang et al., 2022), have been identified as potentially efficient recombinant vaccines. However, there are no reports on the immunoprotective efficacy of VPS against coccidiosis. VPS proteins, which function in membrane protein secretion and sorting, are widely present in the cytoplasm of the apicomplexan parasite cells. Our results also show that the VPS29 protein is highly expressed in *E. tenella* sporozoites. To determine the efficacy of *E. tenella* VPS29, the recombinant rEtVPS29 protein was used in an animal protection assay. Our results showed that rEtVPS29 induced a specific immune response and may be a valuable candidate for novel vaccines against *E. tenella* infection in chickens.

## 5 Conclusion

The EtVPS29 gene fragment from *E. tenella* was cloned and prokaryotically expressed to obtain the recombinant protein rEtVPS29. EtVPS29 protein was found to be widely distributed in the cytoplasm of *E. tenella* sporozoites by immunofluorescence and transgenic overexpression assays. Furthermore, rEtVPS29 induced a specific immune response and provided mild immune protection against *E. tenella* challenge. Thus, EtVPS29 is a potential candidate for the development of novel vaccines against coccidiosis in chickens. However, its functions in parasitic cell integrity, secretory organelle biogenesis, and host infection require further study.

## Data availability statement

The original contributions presented in the study are included in the article/supplementary material. Further inquiries can be directed to the corresponding author.

## Ethics statement

The animal study was reviewed and approved by Animal Care Committee of Zhejiang Academy of Agricultural Sciences.

## Author contributions

T-yS and L-lH conceived and supported the study, T-yS and T-EL wrote the manuscript, YH, T-eL, H-cS, YF and T-yS performed the experiments, T-yS, L-lH and W-cY analyzed the data. All authors contributed to the article and approved the submitted version.
